# Development and Formulation of the Experimental Dentifrice Based on *Passiflora mollissima* (Tumbo) with and without Fluoride Anion: Antibacterial Activity on Seven Antimicrobial Strains

**DOI:** 10.1155/2019/9056590

**Published:** 2019-06-13

**Authors:** Frank Mayta-Tovalino, Eloy Gamboa, Richard Sánchez, Jorge Rios, Ramín Medina, Martín García, Jhonn Asencios

**Affiliations:** ^1^Professor of Stomatology School, Universidad Privada San Juan Bautista, Lima, Peru; ^2^Department of Basic Sciences, Faculty of Health Sciences, Universidad Privada San Juan Bautista, Lima, Peru; ^3^Department of Chemistry, Faculty of Health Sciences, Universidad Privada San Juan Bautista, Lima, Peru; ^4^Stomatology School, Universidad Privada San Juan Bautista, Lima, Peru

## Abstract

**Objectives:**

To develop and formulate a new experimental dentifrice with and without fluoride based on the peel and the fruit of the *Passiflora mollissima* (tumbo) and also to evaluate its antimicrobial activity against seven bacterial strains.

**Methods:**

The sample was calculated using the mean comparison formula, obtaining wells (*n* = 12) for each of the strains evaluated: *S. mutans*, *E. faecalis*, *Actinomyces*, *Lactobacillus*, *C. albicans*, *S. sanguinis*, and *S. oralis*. The antibacterial activity of the dentifrice was evaluated by the direct contact technique.

**Results:**

It was found that the highest antimicrobial activity was only present in pulp-based dentifrices against strains of *S. mutans* 21.0 ± 1.8, *E. faecalis* 16.3 ± 3.9, *Actinomyces* 22.1 ± 1.3, and *Lactobacillus* 21.0 ± 1.3. However, in comparison with other strains such as *C. albicans*, *S. sanguinis*, and *S. oralis*, the peel-based dendrifrice of *Passiflora mollissima* obtained the highest antimicrobial activity.

**Conclusion:**

The experimental dentifrice based on *Passiflora mollissima* had an antibacterial effect against the seven microbial strains during the first 24 and 48 hours.

## 1. Introduction

Toothpastes have been in use for many decades [1], and they are one of the main irreplaceable hygiene products in oral health. The design of toothpaste formulations began in China and India, during 300–500 BC. During that period, crushed bone, powdered egg shell, and clam shells were used as abrasives for oral cleansing. Modern formulations of toothpaste date back to the 19th century; subsequently, soaps and other abrasives were incorporated into these formulations. After 1945, several advances in the formulation of different detergents had been initiated, and sodium lauryl sulfate was used as an emulsifying agent [2, 3]. However, in recent years, the focus has shifted towards the release of active ingredients from natural resources immersed in the formulation to prevent and/or treat certain oral diseases [4].

Oral hygiene is controlled by toothpaste with the aim of reducing oral bacterial flora. Biofilm [1] is a layer that is usually formed on the surface of a tooth. So, some previous studies have shown that dental plaque can be controlled by the physical elimination of bacteria and the use of antimicrobial toothpastes and mouth rinses. There are several types of bacteria in the mouth; some are useful and others are harmful such as *Neisseria*, *Staphylococcus*, *S. pneumoniae*, and *Porphyromonas gingivalis* [5, 6].

On the contrary, the consumption of tropical fruits by the world population is increasing as their nutritional value is better known. These fruits in which the presence of exotic characteristics and nutrients helps treating certain diseases offer an opportunity for local farmers to access special markets. Apart from the nutritional and therapeutic value, most of these native plants have considerable amounts of micronutrients, such as minerals, fibers, vitamins, and secondary phenolic compounds, that can be used in dentistry [7–9].

Tumbo is a plant that belongs to the family Passifloraceae, which grows in Andean areas of South America approximately between 2000 and 3600 meters above the sea level. This native fruit is a rich source of vitamin C, which gives it an excellent antioxidant capacity [9]. It is a source of calcium, iron, phosphorus, potassium, and fiber. In addition, some research on its leaf, skin, and the edible part of this fruit revealed that it is rich in natural phenolic antioxidants. For example, in this sense, some authors have discovered that the antioxidant activity of *Passiflora mollissima* fruit can be compared with that of ascorbic acid, which is the main source of natural antioxidant used in the food industries. On the contrary, its health-promoting effect can be attributed to its polyphenols, especially flavonoids and carotenoids, which have been strongly associated with antioxidant capacity, in addition to presenting certain antimicrobial properties [10–13].

Therefore, the objective of this research was to develop and formulate an experimental dentifrice based on the peel and fruit of *Passiflora mollissima* (tumbo) and to evaluate its antimicrobial effectiveness against seven strains of the oral cavity.

## 2. Materials and Methods

### 2.1. Sample Size

The unit of analysis was formed by a well created on the agar in which the experimental dentifrice based on the fruit and peel of *Passiflora mollissima* (tumbo) was introduced, confronted in the Petri dishes, and inoculated with the cultivated strains of *S. mutans*, *E. faecalis*, *Actinomyces*, *Lactobacillus*, *C. albicans*, *S. sanguinis*, and *S. oralis*. The sample size was calculated by the comparison formula using the Stata® software version 12.0, with an alpha of 0.05 and a power of test of 0.8, obtaining wells (*n* = 12) for each group evaluated. Finally, the following groups were considered based on the peel and pulp of *Passiflora mollissima*:

  Group 1: Petri dishes sown with strains of *Streptococcus mutans* (ATCC® 25175).
  Group 2: Petri dishes sown with strains of *Enterococcus faecalis* (ATCC® 29212).
  Group 3: Petri dishes sown with strains of *Actinomyces* (ATCC® 12104).
  Group 4: Petri dishes sown with strains of *Lactobacillus* (ATCC® 11146).
  Group 5: Petri dishes sown with strains of *Candida albicans* (ATCC® 10231).
  Group 6: Petri dishes sown with strains of *Streptococcus sanguinis* (ATCC® 10556).
  Group 7: Petri dishes sown with strains of *Streptococcus oralis* (ATCC® 6249).
  Group 8: Controls of commercial toothpastes (Dento Herbal®, Colgate Herbal®, Kolynos Herbal®, Sensodyne®, and Parodontax®).

### 2.2. Herbal Classification

To certify the phenotype, the plant was analyzed by the Herbarium of the Natural History Museum of the Universidad Nacional Mayor de San Marcos (UNMSM) with code No. 098-USM-2017, obtaining the following classification:

  Division: Magnoliophyta
  Subclass: Magnoliopsida
  Order: Violales
  Family: Passifloraceae
  Genus: *Passiflora*
  Species: *Passiflora tripartita* var. *mollissima* (Kunth) Holm-Niels and P. Jorg.

### 2.3. Preparation of Tumbo Methanolic Extract

*Passiflora mollissima* was obtained from the city of Huaraz, Peru, located at latitude −9.5277900° and longitude −77.5277800°. To obtain the methanol extract from the pulp, 2000 ml of methanol (CH_3_OH) Merck was used, which was mixed with 2 kg of the “tumbo” pulp. The peel of the fruit, once washed thoroughly with distilled water, was liquefied, and a uniform mass of 1100 g was obtained. Subsequently, the solvent medium, 1000 ml of methanol, was added. Both the pulp and peel mixtures were allowed to stand for a period of 10 days, shaken periodically three times a day, and subjected to multiple filtering processes with the Whatman filter paper. Subsequently, the extracts were placed in a “Memmert” laboratory stove (model EU 500) for a period of 8 days at a constant temperature of 45°C, with the intention of volatilizing ethanol and obtaining highly pure extracts [14, 15].

### 2.4. Sowing Technique

The biotube containing the lyophilized strain of the bacteria belonging to the genus *Streptococcus* was initially taken. This biotube is subjected to a slight pressure at its upper end with the intention of breaking an inner bulb which releases the bacterial suspension in an internal aqueous agent so that a solution containing the strain itself was obtained ready to grow. With the help of a sterile swab, a sample of this solution containing the bacterial strain was taken, to be later taken to the Petri dishes of dimensions 150 × 30 mm containing the culture media TSA agar and nutritive agar; in both cases, they are cultivated with the method of sowing on grass. Once the plantings were completed, they were plated and labeled, and finally, all the cultivated plates were place in a laboratory Auto-Flow UN-4950 incubator at a temperature of 37°C for a period of 24 hours. Finally, the inhibition halos produced by the different evaluated groups were measured by the technique of direct contact in the well, measuring the zones of inhibition in millimeters with a digital Vernier caliper [14, 15].

### 2.5. Preparation of Tumbo Dentifrice (TD)

Precipitated calcium carbonate was used and mixed in 50 ml of distilled or deionized water with continuous stirring, and then the additives of tetrasodium pyrophosphate, AEROSIL (hydrophilic pyrogenic silica) were added: Colloidal silicon dioxide USP, NIPAGIN (methylparaben sodium) or methyl parahydroxybenzoate, saccharin sodium powder, and menthol crystals were also added. The stirring was maintained, and the glycerin was added to achieve a homogeneous mixture; finally, the xanthan gum, titanium dioxide (dye), sodium lauryl sulfate (SLE), and methanol extract of dilute *Passiflora mollissima* pulp (10 g in 10 mL of distilled water) were added [4, 6]. The same procedure was carried out for the dentifrice based on “tumbo” peel in both versions; the only difference was that in one formulation, fluoride was added, and in another, it was not (Table 1).

### 2.6. Declaration of Ethics

Authorization was obtained from the Ethics Committee of the San Juan Bautista Private University (Code CEPB-FCS0007) for the execution of the research project because it forms part of a research line of the university. Because it is an experimental in vitro research, no risks are anticipated, and all the Petri dishes inoculated with the different strains were duly sterilized and discarded under the biosecurity protocols of the laboratory.

### 2.7. Statistical Analysis

For the descriptive analysis of the quantitative variable antimicrobial effect, we proceeded to obtain the descriptive statistics (mean and standard deviation) of all the groups evaluated. Then, the assumption of normality and homogeneity of the variances was determined by the Shapiro–Wilk and Levene tests, respectively. Finally, for the bivariate analysis, the Student *t*-test, the Mann–Whitney *U* test, and the Kruskal–Wallis test were used. A level of significance of *p* < 0.05 was established, and the complete analysis was carried out using the Stata 12.0 software.

## 3. Results

### 3.1. Antimicrobial Activity of Dentifrice of *Passiflora mollissima* (Tumbo) without Fluoride Anion

Table 2 shows that when comparing the antimicrobial effects of the experimental dentifrice of the pulp and peel of the *Passiflora mollissima*, it was evidenced that the pulp of the fruit had the highest activity at 24 or 48 hours against the strains of *S. mutans* (20.0 ± 2.1 mm), *E. faecalis* (15.5 ± 1.1 mm), *Actinomyces* (23.6 ± 4.4 mm), *Lactobacillus* (20.8 ± 0.6), and *C. albicans* (24.7 ± 1.3 mm) of inhibition halos. However, toothpaste based on “tumbo” peel only had a greater effectiveness against strains of *S. sanguinis* (15.9 ± 0.4 mm) and *S. oralis* (16.5 ± 0.4 mm). Despite these differences, both pulp- and peel-based toothpastes presented optimal antimicrobial effects against the seven oral strains evaluated. Finally, when assessing normality, only strains of *E. faecalis*, *Lactobacillus*, *C. albicans*, and *S. sanguinis* showed normal distribution (*p* > 0.05).

### 3.2. Antimicrobial Activity of Dentifrice of *Passiflora mollissima* (Tumbo) with Fluoride Anion

Table 3 shows that when comparing the antimicrobial activity, pulp-based toothpaste with fluoride anion added in the formulation had the highest activity against strains *S. mutans* (21.0 ± 1.8 mm), *E. faecalis* (16.3 ± 3.9 mm), *Actinomyces* (22.1 ± 1.3 mm), *Lactobacillus* (21.0 ± 0.3 mm), and *C. albicans* (21.2 ± 1.9) millimeters of inhibition halos; even this increase in its effectiveness is slightly greater than the activity described in the previous table, so there would apparently be a synergism that generates the fluoride anion in the antimicrobial activity. On the contrary, peel-based toothpaste with the added fluoride ion added only a higher activity against *S. sanguinis* (15.5 ± 0.5 mm) and *S. oralis* (17.0 ± 0.4 mm) strains. Finally, when assessing normality, only strains of *Lactobacillus* and *S. sanguinis* showed normal distribution (*p* > 0.05).

### 3.3. In Vitro Antimicrobial Activity of New “Tumbo” Dentifrice against Commercial Toothpastes

First, when comparing the antimicrobial activity of our new toothpaste based on pulp and tumble skin, no statistically significant differences were found in strains of *S. mutans*, *E. faecalis*, *Actinomyces*, *S. sanguinis*, and *S. oralis* (*p* > 0.05). However, only a statistically significant difference between the effect of the peel and the pulp was observed in the strains of *Lactobacillus* and *C. albicans*. Second, when comparing with commercial herbal dentifrices Dento Herbal®, Colgate Herbal®, Kolynos Herbal®, Sensodyne®, and Parodontax®, statistically significant differences were found *p* ≤ 0.001. Parodontax toothpaste is one of the most active dentrifrices presented together with our experimental dentifrice; however, Sensodyne was the toothpaste that presented the least antimicrobial activity against the seven strains evaluated in this research (Table 4).

## 4. Discussion

Oral diseases are considered a very important community health problem throughout the world. These lesions can be chronic or acute, often requiring not only therapeutic but also preventive treatment. Therefore, the uses of antimicrobial substances for treatment require the use of a drug that has sufficient efficacy at the site of action and that has no side effects [16]. The toothpaste is a mixture of components that are used for cleaning and polishing the teeth. Basically, there are two types of dentifrices, one is simply a preventive dentifrice and the other is therapeutic dentifrice that has certain analgesic, anti-inflammatory, and other components that will help manage certain oral lesions [17]. However, currently, new therapeutic options are being sought in native plants that may offer better clinical benefits in relation to conventional treatments.

Dental products are available in paste, gel, or powder. These are usually applied with a toothbrush to clean the teeth and thus maintain oral hygiene by eliminating the pathogenic microbial flora. Traditionally, toothpastes contain a mild abrasive, detergent, flavoring agent, fluoride, and binder. Other common ingredients include moisturizers, desensitizers, and various medications to prevent various oral pathologies [18, 19]. Toothpaste and mouthwash should contain certain antimicrobial agents that are commonly used as products that improve oral hygiene. Its use goes back to ancient times and continues until now; therefore, there is a need to find new substitutes based on natural products that help prevent diseases of the oral cavity [20].

There is little literature that evaluated the antibacterial properties of *Passiflora mollissima* in its dentifrice form; however, some close studies are consistent with the results of the present study. Although they focus more on evaluating the physical-chemical properties, they conclude that the average abrasivity of formulations with hydrated silica is more than that of formulations with dicalcium phosphate. In addition, a study on 26 commercial toothpastes indicated that the roughness of toothpastes with hydrated silica abrasive was significantly different from that of the toothpaste containing calcium phosphate [21].

On the contrary, some research studies that have studied the antimicrobial effect of new formulations of herbal toothpastes with other different plants to *Passiflora mollissima* (tumbo) mentioned that the antibacterial activity of the dental gel was carried out by the disc diffusion method Assessing its effectiveness against Gram-negative bacteria such as *Pseudomonas aeruginosa* and *E. coli* and Gram-positive bacteria such as *Staphylococcus aureus* incubated for 24 hours at 37°C. When contrasting, these results were similar with our study because we also found a great antimicrobial activity against Gram-positive and Gram-negative germs [17–24].

In the present investigation, comparatively equal and better results have been observed with formulations manufactured in the laboratory that are commercialized daily. Both preparations have shown equal efficacy in terms of antimicrobial activity, capacity, and pH. The comparison of this activity of the marketing pastes with the formulations made in the laboratory suggests that the formulations made in laboratories have a higher sensitivity than the pastes sold. Also, they mention a significant result for cleaning capacity, saying that it is similar to the results obtained in commercial formulations [20–25].

Some investigations describe that toothpastes should have good consistency and smooth texture without signs of deterioration, such as phase separation, gasification, and fermentation, when all samples are placed at a temperature of 45 ± 20°C for a period of 28 days. This confirms that all experimental toothpastes have good stability [4]. There is no literature on formulation of toothpastes of this natural resource; however, when contrasting the methodology of the formulation of the herbal toothpaste, it was found that some studies were done based on the leaves of neem and guava and the bark of cinnamon and that in the formulation testing phase, there are classic problems such as lack of homogeneity, spreadability, and foamability. But, that research showed that herbal toothpaste was greenish brown and showed good homogeneity with absence of bulk and good antimicrobial activity similar to our proposal based on *Passiflora mollissima* [22].

On the contrary, the oral microorganisms that generally produce lactic acid eliminate the mineral materials of the dental enamel leading to the formation of caries and also to the development of infections in the lower layers of the tooth. Although damage and decomposition is a complicated multifactorial disease, the patient's oral hygiene rate is one of the most important factors in the decomposition process. If optimal oral hygiene is provided, there will be no bacterial plaque [23]. Hence, the addition of fluoride in toothpastes reduces tooth decay, so one of the main objectives of this research was to demonstrate the antimicrobial efficacy of the experimental toothpaste based on *Passiflora mollisima* with and without the addition of fluoride anion to verify if this chemical compound promotes a synergism in its antimicrobial effectiveness [24].

Our results showed that the dentifrice based on “tumbo” had an antimicrobial activity against *S. mutans* 21.0 ± 1.8 mm, *E. faecalis* 16.3 ± 3.9 mm, *Actinomyces* 22.1 ± 1.3 mm, and *Lactobacillus* 21.0 ± 1.3 mm; this coincided with the results described by Dave et al. who found an area of inhibition of herbal toothpaste based on *Eugenia caryophyllus*, *Acacia nilotica*, and *Mimusops elengi* against *S. aureus* and *E. coli*. Another study with which we agreed was that carried out by Ghelichli who mentioned that the herbal toothpaste “*Salvadora*” had antibacterial properties against the oral microbial flora is due to the alkaloids present in the stem of the plant “*Salvadora*.” Although the natural resources were different from the *Passiflora mollissima* used in the present study, it is evident that the plants have a great antimicrobial potential against different oral microorganisms [25–27].

The main limitations of this research were as follows: One of the main limitations was the availability of oral microorganisms, because these strains had to be imported from the USA, which demanded a delay in the microbiological tests. Another important limitation was the limited available evidence that evaluated or created an experimental toothpaste based on *Passiflora mollissima*, which made it difficult to analyze and contrast the promising results obtained in this research.

Finally, the present investigation was of social importance given that the patients will probably benefit from demonstrating the antimicrobial effectiveness of this new toothpaste based on *Passiflora mollissima* “tumbo” which is consumed very frequently in the high Andean areas of Peru. On the contrary, it had a theoretical importance because there is scarce literature that has evaluated the therapeutic properties of this natural resource in relation to its use in the stomatology. Finally, it had methodological importance because a new chemical formulation was proposed for the development of this new dentifrice based on “tumbo” which represents a milestone in the Peruvian stomatology that always seeks to use new active ingredients that help prevent and treat certain oral diseases.

## 5. Conclusions

The experimental dentifrice based on the pulp of *Passiflora mollissima* showed to have the greatest antimicrobial effect in comparison with the dentifrice based on the peel, although some antimicrobial strains such as *Actinomyces*, *C. albicans*, *S. sanguinis*, and *S. oralis* were more susceptible to the dentifrice based on the peel. Finally, according to our results, it is concluded that in relation to antimicrobial comparisons with commercial toothpastes, our dental proposal is equal to or superior to the effectiveness of these, which opens a great line of research in relation to this Peruvian natural resource (tumbo).

## Figures and Tables

**Table 1:** Chemical components of experimental dentifrice based on *Passiflora mollisima* “tumbo.”

	Components	Properties
1	Precipitated calcium carbonate	Abrasive
2	Tetrasodium pyrophosphate	Additives
3	AEROSIL (hydrophilic pyrogenic silica)	Additives
4	Colloidal silicon dioxide USP	Preservative
5	NIPAGIN (methylparaben sodium) or methyl parahydroxybenzoate	Sweetener
6	Saccharin sodium, powder or crystallized	Flavoring agent
7	Menthol mint aroma crystals	Moisturizers are added slowly to homogenize the preparation
8	Deionized, demineralized, or distilled water	Moisturizers are added slowly to homogenize the preparation
9	Glycerin (or glycerol)	Binder
10	Xanthan gum	Whiteness to dentifrices
11	Titanium dioxide (dye)	Surfactants
12	Sodium lauryl sulfate (SLE)	1450 ppm fluorine
13	Sodium fluoride (fluoride)	Moisturizers
14	Diluted methanol extract (10 g in 10 mL of distilled water)	Flavoring agent

**Table 2:** Comparison of the antimicrobial activity of tumbo dentifrice against seven strains of the oral cavity (formulation without fluoride).

Microorganisms	Part of the plant	Evaluation time (hours)	Mean ± SD	Min	Max	*p* ^*∗*^
*S. mutans*	Pulp	24	20.0 ± 2.1	17.8	25.2	0.093
		48	19.3 ± 1.1	17.6	21.1	0.479
	Peel	24	16.0 ± 1.5	14.6	18.6	0.008
		48	15.5 ± 1.5	13.8	18.0	0.061

*E. faecalis*	Pulp	24	15.5 ± 1.1	13.8	17.3	0.492
		48	15.2 ± 1.2	13.0	17.0	0.999
	Peel	24	15.5 ± 0.4	15.0	16.5	0.647
		48	15 ± 0.4	14.3	15.8	0.988

*Actinomyces*	Pulp	24	18.3 ± 1	17.3	20.2	0.012
		48	23.6 ± 4.4	17.0	29.0	0.078
	Peel	24	18.7 ± 3.3	16.3	29.1	*p* ≤ 0.001
		48	18.6 ± 3.3	16.3	29.1	*p* ≤ 0.001

*Lactobacillus*	Pulp	24	20.8 ± 0.6	19.8	22.1	0.623
		48	20.2 ± 0.7	18.9	21.5	0.491
	Peel	24	17.6 ± 0.2	17.2	18.1	0.842
		48	17.1 ± 0.2	16.8	17.6	0.511

*C. albicans*	Pulp	24	15.5 ± 0.9	13.8	17.1	0.730
		48	24.7 ± 1.3	23.0	27.2	0.540
	Peel	24	19.3 ± 1.7	16.2	22.3	0.534
		48	18.8 ± 1.8	16.0	22.1	0.274

*S. sanguinis*	Pulp	24	14.9 ± 0.1	14.7	15.3	0.331
		48	14.1 ± 0.5	12.9	14.9	0.555
	Peel	24	15.9 ± 0.4	15.3	16.6	0.447
		48	15.1 ± 0.5	14.2	16.0	0.406

*S. oralis*	Pulp	24	14.5 ± 0.9	13.6	16.4	0.016
		48	14.1 ± 0.9	13.0	16.2	0.195
	Peel	24	16.5 ± 0.4	15.6	17.3	0.699
		48	15.7 ± 0.6	14.8	16.8	0.941

All measurements were made in mm. ^*∗*^Shapiro–Wilk test.

**Table 3:** Comparison of the antimicrobial activity of tumbo dentifrice against seven strains of the oral cavity (formulation with fluoride).

Microorganisms	Part of the plant	Evaluation time (hours)	Mean ± SD	Min	Max	*p* ^*∗*^
*S. mutans*	Pulp	24	21.0 ± 1.8	19.2	25.6	0.029
		48	19.8 ± 1.4	17.9	22.3	0.178
	Peel	24	14.9 ± 0.4	13.8	15.5	0.070
		48	14.5 ± 0.4	13.5	15.2	0.178

*E. faecalis*	Pulp	24	16.3 ± 3.9	12.9	25.3	*p* ≤ 0.001
		48	13.8 ± 1.4	11.3	15.8	0.442
	Peel	24	14.3 ± 0.3	13.8	14.9	0.205
		48	13.9 ± 0.5	12.8	14.8	0.754

*Actinomyces*	Pulp	24	19.0 ± 0.8	17.8	20.2	0.838
		48	22.1 ± 1.3	19.9	24.6	0.717
	Peel	24	18.9 ± 3.2	17.2	29.2	*p* ≤ 0.001
		48	18.9 ± 3.2	17.2	29.2	*p* ≤ 0.001

*Lactobacillus*	Pulp	24	21.0 ± 0.3	20.6	21.9	0.268
		48	20.3 ± 0.5	19.6	21.2	0.795
	Peel	24	18.1 ± 0.4	17.5	18.9	0.704
		48	17.5 ± 0.4	16.8	18.2	0.765

*C. albicans*	Pulp	24	17.4 ± 1.4	15.7	19.7	0.155
		48	21.2 ± 1.9	17.0	23.6	0.180
	Peel	24	21.1 ± 0.9	20.2	22.6	0.064
		48	19.4 ± 3.1	9.8	22.0	*p* ≤ 0.001

*S. sanguinis*	Pulp	24	15.3 ± 0.3	15.0	16.0	0.205
		48	14.0 ± 0.3	13.7	14.9	0.057
	Peel	24	15.5 ± 0.5	14.9	16.5	0.084
		48	14.8 ± 0.5	14.0	16.2	0.126

*S. oralis*	Pulp	24	14.4 ± 0.4	13.6	15.0	0.625
		48	14.3 ± 0.7	12.9	15.5	0.932
	Peel	24	17.0 ± 0.4	16.5	17.7	0.165
		48	16.4 ± 0.5	15.6	17.0	0.029

All measurements were made in mm. ^*∗*^Shapiro–Wilk test.

**Table 4:** Comparison of the antimicrobial activity of tumbo dentifrice against different commercial toothpastes.

Microorganisms	Groups	Mean ± SD	Min	Max	*p* ^*∗*^	*p*	*p*
*S. mutans*	Pulp	20.0 ± 2.1	17.8	25.2	0.093	0.651^++^	*p* ≤ 0.001^†^
	Peel	16.0 ± 1.5	14.6	18.6	0.008		
	Dento Herbal®	17.0 ± 0.6	16.2	17.8	0.734	*p* ≤ 0.001	
	Colgate Herbal®	16.9 ± 0.8	16.1	17.8	0.386		
	Kolynos Herbal®	15.1 ± 0.2	14.9	15.5	0.392		
	Sensodyne®	11.2 ± 0.8	10.0	12.0	0.191		
	Parodontax®	30.2 ± 1.5	28.9	32.5	0.194		

*E. faecalis*	Pulp	15.5 ± 1.1	13.8	17.3	0.492	0.906^+^	*p* ≤ 0.001^*∗∗*^
	Peel	15.5 ± 0.4	15.0	16.5	0.647		
	Dento Herbal®	12.8 ± 0.4	12.3	13.2	0.303	*p* ≤ 0.001	
	Colgate Herbal®	13.9 ± 0.8	12.9	14.9	0.900		
	Kolynos Herbal®	14.2 ± 0.6	13.5	14.9	0.513		
	Sensodyne®	10.6 ± 0.3	10.2	11.0	0.408		
	Parodontax®	15.7 ± 0.2	15.5	16.1	0.649		

*Actinomyces*	Pulp	18.3 ± 1	17.3	20.2	0.012	0.143^++^	*p* ≤ 0.001^†^
	Peel	18.7 ± 3.3	16.3	29.1	0.000		
	Dento Herbal®	14.1 ± 0.4	13.8	14.7	0.303	*p* ≤ 0.001	
	Colgate Herbal®	15 ± 0.1	14.8	15.2	0.714		
	Kolynos Herbal®	13.4 ± 0.1	13.2	13.6	0.000		
	Sensodyne®	13.2 ± 0.2	12.9	13.5	0.000		
	Parodontax®	14.1 ± 0.5	13.5	14.9	0.889		

*Lactobacillus*	Pulp	20.8 ± 0.6	19.8	22.1	0.623	*p* ≤ 0.001^+^	*p* ≤ 0.001^*∗∗*^
	Peel	17.6 ± 0.2	17.2	18.1	0.842		
	Dento Herbal®	17.7 ± 0.6	17.0	18.3	0.443	*p* ≤ 0.001	
	Colgate Herbal®	21.2 ± 0.9	20.1	22.3	0.997		
	Kolynos Herbal®	22.1 ± 0.8	20.9	23.0	0.696		
	Sensodyne®	15.5 ± 2.8	12.9	18.3	0.126		
	Parodontax®	15.2 ± 0.2	14.9	15.5	0.910		

*C. albicans*	Pulp	17.4 ± 1.4	15.7	19.7	0.730	*p* ≤ 0.001^+^	*p* ≤ 0.001^†^
	Peel	21.1 ± 0.9	20.2	22.6	0.534	*p* ≤ 0.001	
	Dento Herbal®	17.9 ± 0.6	17.3	18.7	0.513		
	Colgate Herbal®	21.7 ± 0.4	21.2	22.3	0.970		
	Kolynos Herbal®	20.9 ± 1.5	19.9	23.2	0.025		
	Sensodyne®	0	0	0	—		
	Parodontax®	24.4 ± 0.6	23.8	25.0	0.111		

*S. sanguinis*	Pulp	14.9 ± 0.1	14.7	15.3	0.331	1.000^+^	*p* ≤ 0.001^*∗∗*^
	Peel	15.9 ± 0.4	15.3	16.6	0.447		
	Dento Herbal®	19.7 ± 0.5	19.0	20.2	0.369	*p* ≤ 0.001	
	Colgate Herbal®	21.1 ± 0.5	20.4	21.8	0.994		
	Kolynos Herbal®	20.4 ± 1.1	19.6	22.1	0.211		
	Sensodyne®	0	0	0	—		
	Parodontax®	26.1 ± 0.5	25.6	26.9	0.680		

*S. oralis*	Pulp	14.5 ± 0.9	13.6	16.4	0.016	0.425^++^	*p* ≤ 0.001^†^
	Peel	16.5 ± 0.4	15.6	17.3	0.699		
	Dento Herbal®	16 ± 0.6	15.2	16.8	0.999	*p* ≤ 0.001	
	Colgate Herbal®	15.6 ± 0.5	15.1	16.3	0.688		
	Kolynos Herbal®	19.9 ± 0.1	19.8	20.2	0.849		
	Sensodyne®	0	0	0	—		
	Parodontax®	17.7 ± 0.4	17.2	18.2	0.420		

All measurements were made in mm. The concentrations were calculated from the dilutions of the active ingredient; the Sensodyne control group was excluded from any statistical analysis because the antimicrobial activity was not present. ^*∗*^Shapiro–Wilk test. ^+^Student *t*-test. ^++^Mann–Whitney *U* test. ^*∗∗*^ANOVA test. ^†^Kruskal–Wallis test. Level of significance *p* < 0.05.

## Data Availability

The data used to support the findings of this study are available from the corresponding author upon request.
